# A Simple and Reliable Protocol for the Preparation and Culturing of Fresh Surgically Resected Human Glioblastoma Tissue

**DOI:** 10.3390/mps3010011

**Published:** 2020-01-22

**Authors:** Liyen Katrina Kan, Katharine J Drummond, Martin Hunn, David A Williams, Terence J O’Brien, Mastura Monif

**Affiliations:** 1 Department of Neuroscience, Monash University, Melbourne, VIC 3004, Australia; liyen.kan@monash.edu (L.K.K.); Terence.OBrien@monash.edu (T.J.O.); 2 Department of Neurology, Royal Melbourne Hospital, Melbourne, VIC 3050, Australia; 3 Department of Physiology, The University of Melbourne, Melbourne, VIC 3010, Australia; davidaw@unimelb.edu.au; 4 Department of Surgery, The University of Melbourne, Melbourne, VIC 3010, Australia; Kate.Drummond@mh.org.au; 5 Department of Neurosurgery, Royal Melbourne Hospital, Melbourne, VIC 3050, Australia; 6 Department of Neurosurgery, Alfred Health, Melbourne, VIC 3004, Australia; m.hunn@alfred.org.au

**Keywords:** brain tumour, glioma, glioblastoma, cell culture, tissue culture, primary cell culture

## Abstract

Glioblastoma is a heterogeneous glial cell malignancy with extremely high morbidity and mortality. Current treatment is limited and provide minimal therapeutic efficacy. Previous studies were reliant on cell lines that do not accurately reflect the heterogeneity of the glioma microenvironment. Developing reliable models of human glioblastoma is therefore essential. Direct culture of human brain tumours is often difficult and there is a limited number of protocols available. Hence, we have developed an effective method for the primary culture of human glioblastoma samples obtained during surgical resection. Culturing tumour tissue direct from human brain is advantageous in that cultures (1) more closely resemble true human disease, relative to the use of cell lines; (2) comprise a range of cellular components present in the natural tumour microenvironment; and (3) are free of added antibodies and reagents. Additionally, primary glioblastoma cultures are valuable in studies examining the effects of anti-cancer pharmaceuticals and therapeutic agents, and can be further used in live cell imaging, immunocytochemistry, flow cytometry and immunoassay experiments. Via this protocol, cells are maintained in supplemented medium at 37 °C (5% CO_2_) and are expected to achieve sufficient confluency within 7 days of initial culture.

## 1. Introduction

Glioblastoma is the most predominant and aggressive central nervous system malignancy, accounting for over 60% of all brain tumours in adults [1]. Current treatments are limited in range and efficacy, with patients surviving for a median of only 14 to 15 months post-diagnosis [1]. Hence, a deeper understanding of glioblastoma pathology and the mechanisms of tumour pathogenesis is pivotal for research progression. The heterogeneity of glioblastoma is reflected by the crosstalk between tumour cells, microglia and a multitude of invading immune cells and cytokines [2,3]. This presents as a hurdle to overcome when developing reliable models of glioblastoma.

Conventionally, glioblastoma research has been based on data obtained from homogeneous, murine and patient-derived cell lines. While cell lines provide a rapid and reproducible means of *in vitro* experimentation [4], they do not accurately mimic the heterogeneity of tumours *in vivo*. Glioblastoma cell lines have been demonstrated to express markedly different gene expression profiles when compared to primary tumours [5,6] and are susceptible to genetic drift across passages that alter experimental reproducibility [4].

Importantly, surgically resected human glioblastoma tissue retains the molecular and cellular characteristics of the original tumour mass. These samples have also been demonstrated to express microglia, the largest immune cell infiltrates of the glioblastoma microenvironment [7,8]. Other immune cells present may include lymphocytes, neutrophils, monocytes/macrophages and myeloid-derived suppressor cells [9,10,11,12]. Cellular infiltrates serve a range of functions that disparately affect tumour growth. Hence, relative to cell lines, the culturing of samples directly from surgically resected glioblastoma more closely resembles true disease and takes into account the effect of infiltrating immune cells. Existing protocols for the culture of primary glioblastoma tissue are limited and have limited growth efficacy [13]. In light of this, we demonstrate a simple, reliable and efficient protocol for the direct culture of human glioblastoma tissue. Importantly, this protocol processes tumour samples for culturing immediately after surgical resection, which minimizes potential environmental disruptions that may significantly affect the tumour microenvironment. This method is particularly useful for drug testing *in vitro*, and can be used to assess the presence of various cell populations and biomarkers within the tumour microenvironment via immunocytochemistry, flow cytometry and immunoassays.

## 2. Experimental Design


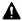
**Ensure ALL materials and equipment are sterile prior to usage**.

### 2.1. Materials

#### 2.1.1. Poly-D-Lysine Plate Coating Solution

- Poly-D-lysine hydrobromide powder, 5 mg (Merck, Australia; Cat. no.: P6407)
- Sterile distilled water

#### 2.1.2. Enzymatic Tissue Dissociation Solution

- Papain from papaya latex (Merck, Australia; Cat. no.: P3125)
- Earle’s Balanced Salt Solution (EBSS; Thermo Fisher Scientific, Australia; Cat. no.: 14155063)

#### 2.1.3. Culture Medium

- Minimum Essential Medium, 1X, 500 mL (Thermo Fisher Scientific, Australia; Cat. no.: 10370021)
- D-glucose (Merck, Australia; Cat. no.: G7021)
- L-glutamine (Thermo Fisher Scientific, Australia; Cat. no.: 25030081)
- Penicillin-streptomycin (Thermo Fisher Scientific, Australia; Cat. no.: 15070063)
- Heat-inactivated fetal bovine serum (Thermo Fisher Scientific, Australia; Cat. no.: 10100147)
- Corning® MITO+ Serum Extender (Merck, Australia; Cat. no.: DLW355006)

### 2.2. Equipment

- Class II biological safety cabinet
- 12-well cell culture plates (Merck, Australia; Cat. no.: SIAL0512)
- 18 mm glass coverslips (Thermo Fisher Scientific, Australia; Cat. no.: CB00180RA020MNT0)
- Paraffin
- Surgical tweezer
- 50 mL syringe (Merck, Australia; Cat. no.: Z683698)
- Syringe filter, 0.2 µm pore (Merck, Australia; Cat. no.: CLS431229)
- 50 mL centrifuge tubes (Merck, Australia; Cat. no.: CLS430828)
- Petri dish (Merck, Australia; Cat. no.: P5481)
- Surgical scalpel
- Pipette Boy (Eppendorf, Australia; Cat. no.: 4430000018)
- 10 mL pipettes (Sterilin, Australia; Cat. no.: 47510)
- Pasteur pipette with rubber bulb
- Bunsen burner
- Water bath set at 37 °C
- Automated cell counter
- Humidified 5% CO_2_/95% O_2_ incubator, 37 °C (Panasonic; Model no.: MCO-170AICUV-PE)

## 3. Procedure


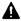
**Ensure ALL experiments are completed under sterile conditions with appropriate aseptic techniques to minimize sample contamination and exposure to human tissue**.

### 3.1. Poly-D-Lysine Plate Coating Time for Completion: 3 Days

1. Add 50 mL of autoclaved distilled water into 5 mg stock poly-D-lysine powder using a 50 mL syringe with a 0.2 µm pore syringe filter. Re-cap the stock bottle and shake lightly.
2. Transfer one 18 mm glass coverslip per well onto 12-well cell culture plates. **NOTE:** A single 50 mL poly-D-lysine solution can be used to prepare approximately thirteen poly-D-lysine-coated 12-well cell culture plates.
3. Add 300 µL of poly-D-lysine solution per well and leave at room temperature (25 °C) for 2 h.
4. Aspirate residual poly-D-lysine solution in each well and leave in sterile conditions at room temperature for 48 h or until the wells have dried.
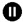
**PAUSE STEP:** Seal plates with paraffin and store at 2–8 °C until usage.

### 3.2. Sample Preparation and Tissue Culture. Time for Completion: 75 Min for Sample Preparation. 7 Days to Reach 80% Confluency

**Figure 1:**
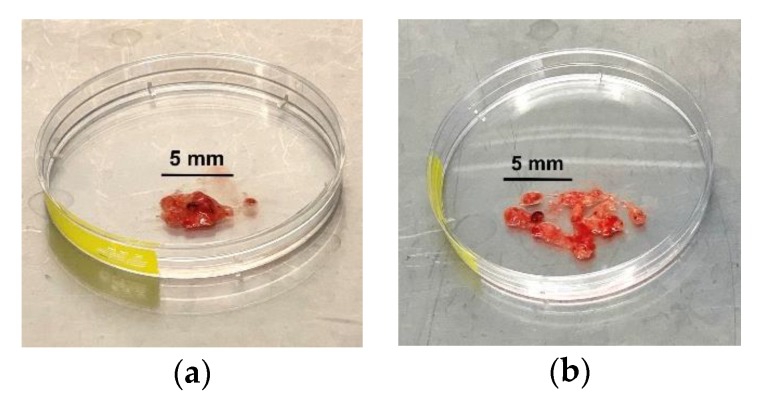
Fresh surgically resected human glioblastoma tissue (**a**) prior to and (**b**) after fragmentation with a surgical scalpel.

6. Pre-warm 10 mL of EBSS in a 50 mL centrifuge tube and culture medium at 37 °C.
7. Immediately after collection of the fresh sample, add 200 units of papain from papaya latex into warm EBSS.
  **NOTE:** This concentration of papain applies to a sample of approximately 5 mm^2^. Increase concentration with larger samples.
8.
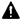
**CRITICAL STEP:** Transfer the tumour tissue into a petri dish and gently fragment the tissue with a surgical scalpel (Figure 1).
9. Transfer fragmented sample into the warm EBSS and papain solution and place in a water bath at 37 °C for 40 min.
10. Gently aspirate sample with a 10 mL pipette and transfer into a new 50 mL centrifuge tube with 3 mL culture medium. Wash in triplicate with culture medium, topping up with new culture medium after each wash.
11.
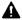
**CRITICAL STEP:** With a gentle up and down pipetting motion, use a Pasteur pipette with a rubber bulb to disassociate the cells, creating a single cell suspension.
  **TIP:** Soften the edges of the Pasteur pipette with a Bunsen burner to minimize disruption to cellular integrity.
12. Count the cells using an automated cell counter and top up accordingly with culture medium to make up 5 × 10^5^ viable (live) cells/mL. Alternatively, a haemocytometer can be used.
13. Homogenize the cell solution by pipetting up and down and transfer 1 mL per well onto 12-well cell culture plates pre-coated with poly-D-lysine. Maintain cells in a humidified incubator at 37 °C with 5% CO_2_ for 7 days or until cells are 80% confluent.
  **NOTE:** For optimal growth, replace the culture medium every 3–4 days.

## 4. Expected Results

It is important to note that due to existing heterogeneity between each tumour, there may be a difference in proliferation rate per culture. The time taken to reach 80% confluency might vary between each culture; we have previously cultured 20 tumours using this protocol, of which 17 (85%) reached 80% confluency within a time span of 7 days.

**Figure 2:**
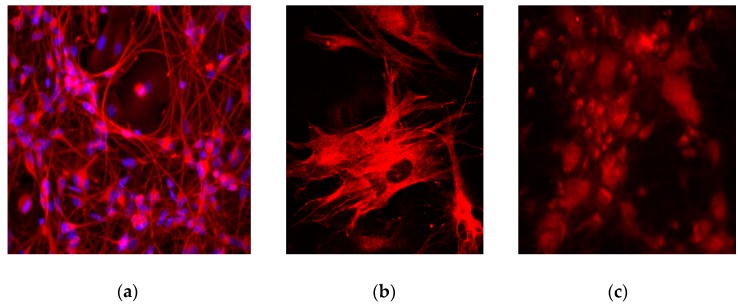
Immunocytochemical staining and fluorescence confocal microscopy of primary human glioblastoma culture fixed at 80% confluency. Glioma cells were stained with a primary anti-glial fibrillary acidic protein (GFAP) antibody and secondary Texas Red X. Cell nuclei were stained with DAPI. (**a**) Stained cultures at 40 × objective. (**b**) Stained cultures at 63 × objective. (**c**) Immune cell stained with anti-CD11b (red) present within a glioblastoma culture imaged with a 40X objective.

Surgically resected human glioblastoma tissue cultured via this protocol closely reflect the cellular heterogeneity of the original tumour. In particular, glioma cells as well as microglia and other immune cells are expected to contribute to the vast majority of the cellular architecture [7,8]. In Figure 2, we show the presence of glioma cells marked by glial fibrillary acidic protein (GFAP), as well as CD11b-positive immune cell infiltrates in culture. CD11b is known to be expressed on the surface of many immune cells, including tumour-associated microglia/macrophages, monocytes, neutrophils and natural killer cells [14,15,16]. Cells in culture were fixed at 80% confluency, stained via immunocytochemistry and visualized using fluorescence confocal microscopy.

### Troubleshooting

Potential issues arising and their respective troubleshooting solutions are presented in Table 1.

## 5. Reagents Setup

### 5.1. Poly-D-Lysine Plate Coating Solution

- Poly-D-lysine hydrobromide powder, 5 mg
- 50 mL sterile distilled water

### 5.2. Enzymatic Tissue Dissociation Solution

- 200 units papain from papaya latex (approximately 155 µL in 10 mL EBSS)
- 10 mL EBSS

### 5.3. Culture Medium

- Minimum Essential Medium, 1 ×, 500 mL
- 1 mM D-glucose
- 2 mM L-glutamine
- 50 units/mL penicillin-streptomycin
- 10% heat-inactivated foetal bovine serum
- Corning® MITO+ Serum Extender (Merck, Australia; Cat. no.: DLW355006)

## Figures and Tables

**Table 1:** Potential issues arising and respective troubleshooting.

Step	Issue	Causes	Suggestions
11	Tissue does not easily dissociate when pipetted	The tissue sample was not sufficiently fragmented in Step 8 The quality/strength of the papain enzyme solution might be compromised The tissue was not left in papain solution for long enough	Ensure the tissue is fragmented into smaller (<2 mm) pieces in Step 8 Ensure proper storage of papain solution when not in use. Ensure that the papain solution has not expired. Increase the concentration of papain with larger samples. Leave sample in papain solution for an additional 5–10 min
13	Larger chunks of tissue are present in homogenized cell solution	Tissue was not properly dissociated in Step 11	Follow the suggestions for Step 11 (above) to ensure tissue is properly dissociated. Use a 10 mL pipette to mix cell solution and allow larger pieces of tissue to settle at the bottom. Exclude the larger pieces of tissue when transferring onto 12-well culture plates. Change culture medium after 3 days.
13	Cells are not becoming confluent	The tissue was damaged by over-fragmentation in Step 8 The tip of the Pasteur pipette was broken or too sharp, which damaged cellular integrity when pipetted The quality of the culture medium has been compromised	Ensure tissue is fragmented gently in Step 8 Smoothen the tip of the Pasteur pipette with a Bunsen burner prior to usage. Ensure tip is not damaged. Ensure proper storage of culture medium when not in use and that the medium or any component of the medium has not expired.
